# Data on the potential impact of food supplements on the growth of mouse embryonic stem cells

**DOI:** 10.1016/j.dib.2016.03.098

**Published:** 2016-04-04

**Authors:** Marcelo Correia, Maria I. Sousa, Ana S. Rodrigues, Tânia Perestrelo, Sandro L. Pereira, Marcelo F. Ribeiro, João Ramalho-Santos

**Affiliations:** a Ph.D. Programme in Experimental Biology and Biomedicine (PDBEB), CNC – Center for Neuroscience and Cell Biology, University of Coimbra, Coimbra, Portugal; b Institute for Interdisciplinary Research (IIIUC), University of Coimbra, Coimbra, Portugal; c Biology of Reproduction and Stem Cell Group, CNC – Center for Neuroscience and Cell Biology, University of Coimbra, Coimbra, Portugal; d Department of Life Sciences, University of Coimbra, Coimbra, Portugal

**Keywords:** Mouse embryonic stem cells, Kaempferol, Berberine, Tauroursodeoxycholic acid, Cell growth, Toxicology, Dietary supplements

## Abstract

The use of new compounds as dietary supplements is increasing, but little is known in terms of possible consequences of their use. Pluripotent stem cells are a promising research tool for citotoxicological research for evaluation of proliferation, cell death, pluripotency and differentiation. Using the mouse embryonic stem cell (mESC) model, we present data on three different compounds that have been proposed as new potential supplements for co-adjuvant disease treatments: kaempferol, berberine and Tauroursodeoxycholic acid (TUDCA). Cell number and viability were monitored following treatment with increased concentrations of each drug in pluripotent culture conditions.

**Specifications Table**

**Table:** 

Subject area	Biology
More specific subject area	Food Toxicology
Type of data	Image (microscopy), graphs
How data was acquired	Microscope, cell count (hemocytometer)
Data format	Analyzed
Experimental factors	Kaempferol, berberine and TUDCA were added to culture mouse embryonic stem cells (mESC) with different concentrations for three days; pictures were acquired and colony size as cell numbers assessed.
Experimental features	200 µM kaempferol induce a significant reduction of mouse embryonic stem cell number. Berberine in a range from 5 to 100 µM also induces a significant reduction in cell number after 3 days in culture. Tauroursodeoxycholic acid – TUDCA [50–250 µM] did not affect embryonic stem cell growth after 3 days in culture.
Data source location	Coimbra, Portugal
Data accessibility	Data is within this article.

**Value of the data**

• Our data provides information about possible citotoxicological effects of different concentrations of kaempferol, berberine and TUDCA on mESC.
• Kaempferol and berberine reduce mESC cell number in *in vitro* culture conditions. TUDCA does not affect cell viability.
• The data may be valuable for new research concerning the evaluation of favorable physiological effects and safety of these compounds as food supplements.

## 1 Data

Taking advantage of embryonic stem cells as a citotoxicological model [1], [2], [3], [4], we monitored cell number and viability after acute exposure of mESCs to kaempferol [1], berberine and Tauroursodeoxycholic acid (TUDCA), potential supplements for co-adjuvant disease treatments.

**Fig. 1:**
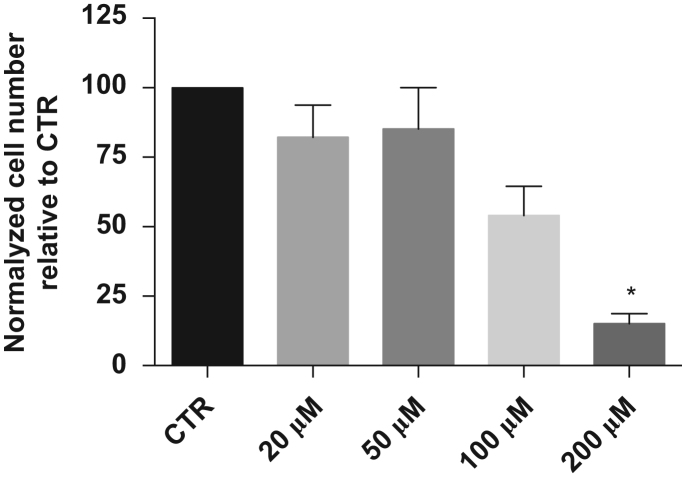
At the start of the experiment, E14Tg2.a mESC were plated at a density of 5000 cells/cm^2^, and allowed to grow for 72 h in the presence of kaempferol in culture media. At 72 h, cells were harvested and total number of cells was counted. Mean±SEM data are represented in histograms of three independent experiments. Statistical significance of the data was considered when *p<0.05 (A).

Data in Fig. 1 shows a reduction in cell number in culture for 100 and 200 μM, with statistical significance for the last concentration (Fig. 1A). For more exhaustive data regarding kaempferol and mESCs, please see [1].

**Fig. 2:**
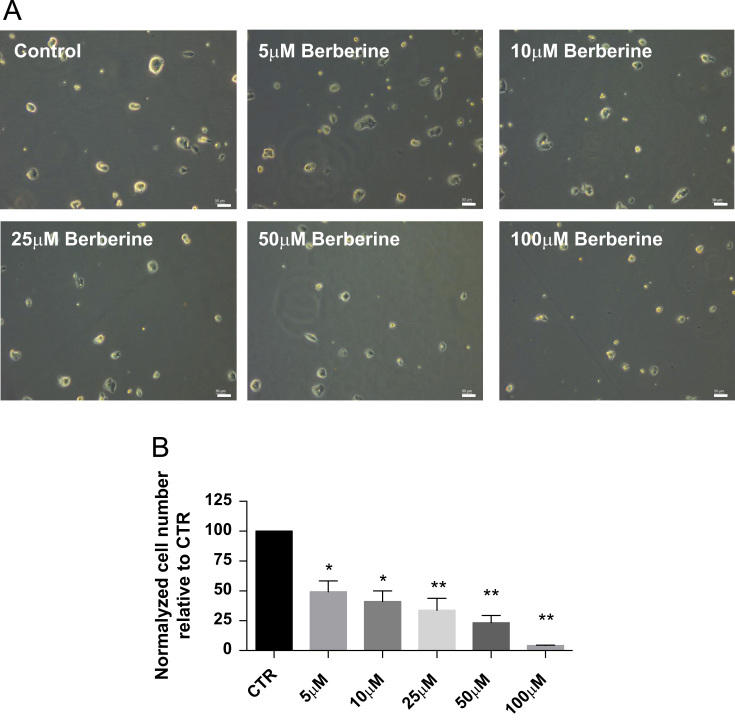
Density of 5000 mESCs/cm^2^ was plated and kept in culture for 72 h, with media and berberine renewal every 24 h. Pictures were acquired from randomly fields using phase contrast microscopy at the end of the experimental procedure (72 h). Scale bar represents 50 μm (A). Cells were harvested at the end of the experimental procedure and the total number of cells was assessed. Mean±SEM data are represented in histograms of three independent experiments. Statistical significance of the data was considered when *p<0.05 and **p<0.01 (B).

Data in Fig. 2 shows a decrease in number and size of mESC colonies incubated with increasing concentrations of berberine [5–100 μM] with no obvious implications in terms of spontaneous differentiation (Fig. 2A). Fig. 2B shows a significant reduction in cell number for all experimental concentrations of berberine on mESC.

**Fig. 3:**
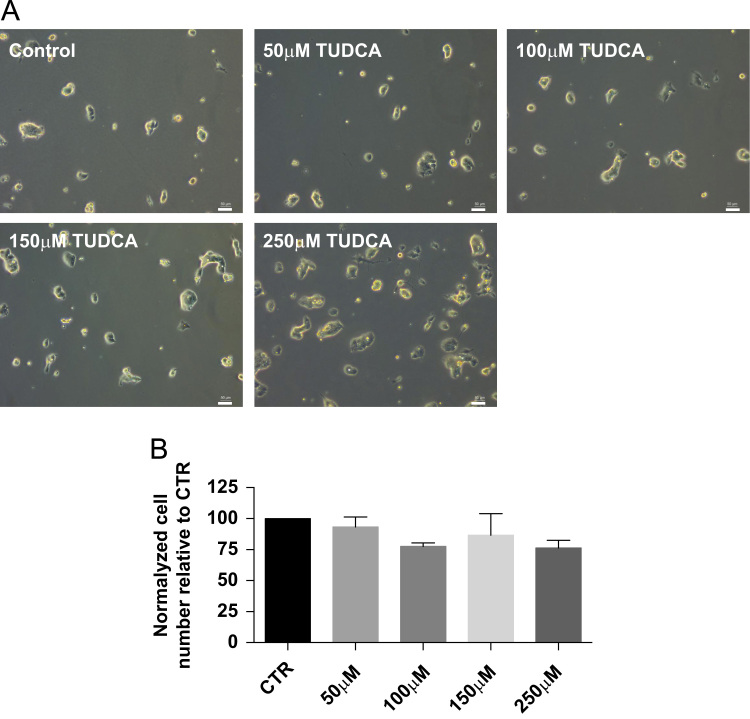
mESCs were plated at a density of 5000 cells/cm^2^ and allowed to growth for 72 h. Fresh media and TUDCA were renewed every 24 h. Pictures were acquired from randomly fields using phase contrast microscopy at the end of the experimental procedure (72 h). Scale bar represents 50 μm (A). Cells were harvested at the end of the experimental procedure and total number of cells was counted. Mean±SEM data are represented in histograms of three independent experiments (B).

Finally, data in Fig. 3 shows that mESC incubated with 250 μM of Tauroursodeoxycholic acid (TUDCA) have larger colonies with few protuberances arising from the edges of the pluripotent colonies, but not for the more reduced concentrations used [50–150 μM] (Fig. 3A). Cell number determination shows no differences after exposure to all concentrations of TUDCA after 72 h (Fig. 3B).

## 2 Experimental design, materials and methods

### 2.1 mESC culture

E14Tg2a mouse embryonic stem cells were kindly provided by Dr. Miguel Ramalho-Santos (University of California, San Francisco, USA). Cells were routinely maintained as previously described [4], [5], [6]. Briefly, mouse embryonic stem cells were allowed to grow for three days, with fresh media change every day. Complete culture medium – KODMEM – is composed of KnockOut-DMEM, 15% KnockOut serum replacement, 2 mM L-glutamine, 100 U/ml penicillin/streptomycin (Life Technologies), 1% non-essential amino acids, 0.1 mM mercaptoethanol (Sigma-Aldrich) and 1.000U of Leukemia inhibitory factor (LIF) – (Chemicon – Millipore) at 37 °C and 5% CO_2_ conditions. At the third day of cell growth, cells were harvested with Accutase (life technologies), centrifuged, counted and plated at a density of 5000 cells/cm^2^. All plates were coated with 0.1% gelatin (Sigma) before cell plating.

### 2.2 Drugs

Kaempferol was purchased from Sigma-Aldrich. A primary stock of 200 μM were prepared in DMSO (Sigma-Aldrich) and stored into freeze aliquots. Each day of media supplementation, new aliquots were used for secondary stock solutions preparation.

Berberine was kindly provided by Dr. Carlos Palmeira (Department of Life Sciences, University of Coimbra). A stock of 50 mM berberine was prepared in DMSO (Sigma-Aldrich) and aliquots were stored at −20 °C. On every daily media supplementation a new aliquot was used for the preparation of secondary stock solutions (1 mM).

TUDCA was kindly provided by Dr. Susana Solá (iMed.ULisboa, Faculty of Pharmacy, University of Lisbon, Lisbon). Every month a fresh working stock of 100 mM TUDCA was prepared in water, aliquoted and stored at 4 °C. On every daily media supplementation, a new aliquot was used.

### 2.3 Experimental design

**Fig. 4:**
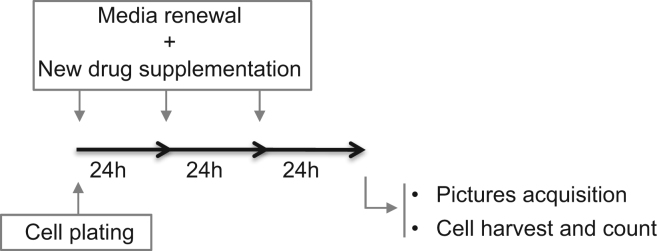
Experimental design.

E14Tg2.a mESCs were grown for 72 h in KODMEM culture media supplemented with the different drugs used at different concentrations, immediately after cell plating. KODMEM media was always supplemented with LIF, a growth factor required for pluripotency maintenance. Culture medium and drugs were renewed every 24 h for three days. At 72 h post-incubation cells were used for (1) acquisition of micrographs and (2) cell counting. The experimental design is represented in Fig. 4.

### 2.4 Image acquisition and cell count

Micrographs were acquired in phase-contrast of a Leica DMI3000B microscope with a 10× objective and a Leica DFC425C camera. Pictures of random fields of the culture dishes were acquired.

For cell count purpose, mESC were gently rinsed with PBS and dissociated with Accutase (Life Technologies) for 5 min at 37 °C. Cells were then collected, centrifuged and a small aliquot was used for staining with Trypan Blue (Sigma-Aldrich). Viable cells were counted with a hemocytometer. Cell number *per* dish was calculated. For data presentation, cell number was normalized for the control situation. Statistical analysis was conducted with total cell numbers.

### 2.5 Data analysis and statistics

Prism 6 (GraphPad) was used to perform statistical analysis. Values represent means±standard error of mean (SEM) of three separate experiments. ANOVA followed by the Bonferroni post-hoc test was used for multiple comparisons between different experimental conditions. Paired-sample statistic was used once all different data acquisitions within the same experiment was performed and collected at the same time. Statistical analysis was performed using raw data and, for a comprehensive analysis, data were normalized to 100% of the control experimental condition. Statistical significance was determined at *p≤0.05 and **p≤0.01.
